# An outbreak of artemisinin resistant falciparum malaria in Eastern Thailand

**DOI:** 10.1038/srep17412

**Published:** 2015-11-30

**Authors:** Mallika Imwong, Thantip Jindakhad, Chanon Kunasol, Kreepol Sutawong, Phisitt Vejakama, Arjen M. Dondorp

**Affiliations:** 1 Department of Molecular Tropical Medicine and Genetics, Faculty of Tropical Medicine, Mahidol University, Bangkok 10400, Thailand; 2 Mahidol-Oxford Tropical Medicine Research Unit, Faculty of Tropical Medicine, Mahidol University, Bangkok 10400, Thailand; 3 Buntharik Hospital, Amphoe Buntharik, Ubon Ratchathani 34230, Thailand; 4 Centre for Tropical Medicine, Nuffield Department of Medicine, University of Oxford, Oxford, United Kingdom

## Abstract

Artemisinin resistant falciparum malaria is an increasing problem in Southeast Asia, but has not been associated with increased transmission of the disease, yet. During a recent outbreak in 2014 in Ubon Ratchatani, Eastern Thailand, parasites from 101 patients with falciparum malaria were genotyped for antimalarial drug resistance markers. Mutations in the Kelch13 marker for artemisinin resistance were present in 93% of samples, mainly C580Y from 2 major clusters as identified by microsatellite typing. Resistance markers for antifolates and chloroquine were also highly prevalent. Most strains (91%) carried single copy number *PfMDR1*, suggesting sustained sensitivity to mefloquine, the partner drug in the local first-line artemisinin combination therapy (ACT). The high prevalence of artemisinin resistance in this recent malaria outbreak suggests but does not prove a causative role in increased transmission. Careful monitoring of ACT efficacy and additional genetic epidemiological studies are warranted to guide the public health response to the outbreak.

The Greater Mekong Sub region has seen impressive gains in malaria control over the last 15 years. This includes Thailand, where the total number of reported cases has gone down from a total of 78,561 cases in 2000 to 33,302 cases in 2013 of which 44% were caused by *P. falciparum* and 47% by *P. vivax*. The corresponding annual parasite incidence (API) is below 0.5/1000 per year, and transmission is confined to its border areas^1^. Malaria elimination has become an official goal, with a target of eliminating the disease from 80% of Thailand by 2020. There are multiple contributors to this positive development, but the wide deployment of the highly effective artemisinin combination therapies (ACTs) since 1995 is thought to have been an important contributor. The artemisinin component in ACTs is potent and fast acting and in addition reduces transmission through its effect on gametocytes, the sexual stages of the parasite. The partner drugs in ACTs are slower acting, but have in contrast with the artemisinins a long plasma half-life. This enables the elimination of the remaining parasites after the recommended 3-day treatment course^2^. Because of their central role in malaria control, the recognition of emerging partial artemisinin resistance in *P. falciparum* on the Cambodian-Thai border in 2008, and its subsequent geographical spread has caused major concern^3^,^4^,^5^,^6^. Initially the much slower clearance of parasitaemia, which defines artemisinin resistance, did not result in falciparum malaria treatment failure with ACTs. However, concomitant emergence and selection of partner drug resistance, including to mefloquine and piperaquine, has resulted in high ACT failure rates on the Cambodian-Thai, the Cambodia-Laos and the Thai-Myanmar border^7^,^8^,^9^. Absence of effective antimalarial treatment in multidrug resistant (MDR) falciparum malaria evidently threatens malaria control, and potentially could render the malaria elimination agenda futile. Southern Laos has witnessed an important increase in falciparum malaria cases since 2012. However, the cause of this outbreak has been attributed to increased employment of cross-country and cross-border migrants in forested areas, rather than increased transmission caused by treatment failure, since its first-line treatment artemether-lumefantrine showed good efficacy in WHO coordinated surveillance studies^10^. More recently, the adjacent province of Ubon Ratchathani in Thailand, which shares borders with Southern Laos and Northern Cambodia, has witnessed an important outbreak of malaria starting in 2014. The number of reported malaria cases went up from 1,201 in 2013 to 7,887 cases in 2014, an increase of 557% and accounting for 26% of all malaria in the country^11^. Of these cases, 44% was caused by *P. vivax* and 31% by *P. falciparum*. Most cases were reported in the age group from 15–24 years with a predominance of males (74%) of which a majority were forest goers. Efficacy of the current first line treatment regimen, a 3-day course of artesunate-mefloquine, in patients affected by the outbreak is currently not known. Drug resistance for some of the antimalarials can be assessed by molecular markers, including Kelch13 propeller region mutations for artemisinins^12^, *Pfmdr1* amplification for mefloquine^13^, DHFR and DHPS mutations for sulphadoxin-pyrimethamine^14^, and PfCRT mutations for chloroquine^15^. Various point mutations in PfMDR1 contribute to lumefantrine, mefloquine, amodiaquine and other antimalarial drug resistance. To assess the implications of the outbreak of falciparum malaria in Ubon Ratchatani it is essential to know whether this is caused by a fast expansion of MDR falciparum malaria. The current study uses molecular markers for antimalarial drug resistance and microsatellite markers for genetic epidemiology to address this.

## Results

### *P. falciparum* resistance marker gene patterns

**Figure 1:**
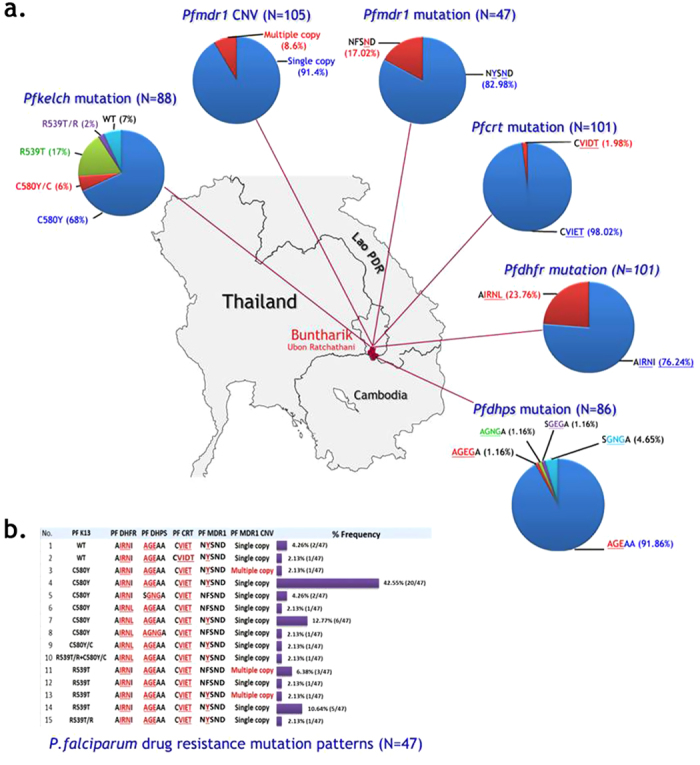
(**a**) Pie charts representing proportions of mutations or gene-amplification in five resistance genes (*Pfkelch*, *pfcrt*, *pfdhfr*, *pfdhps* and *pfmdr1*) observed in *P. falciparum* isolates from Ubon Ratchathani. A total of 101 samples were genotyped, but full genotyping of all resistance markers was not accomplished in all samples (see denominators shown next to pie charts) The map was created using Adobe^®^ Photoshop^®^ CS6 version 13.1.2 × 64 software (Copyright© 1990–2012 Adobe Systems Incorporated). (**b**) Bar chart representing the patterns observed in the five antimalarial drug resistance genes assessed in this study.

Figure 1 provides a summary of the molecular resistance markers identified in this study. A total of 101 patients presenting with uncomplicated falciparum malaria in Buntharik district hospital were studied, but full genotyping of all resistance markers was not accomplished in all samples. Full length sequencing of *P. falciparum PfKelch* gene was assessed in samples from 88 patients presenting with uncomplicated falciparum malaria (Fig. 1A). Point mutations in the propeller region of the gene were found in 82/88 (93%) of samples. The major mutant type was C580Y (65/88: 74%) followed by R539T (17/88: 19%). A small number of samples showed multiple genotypes suggesting polyclonal infections: C580Y/C in 5/88 (6%) and R539T/R in 2/88 (2%) of patient samples.

*Pfmdr1* copy number assessment showed amplification of the gene in 9/101 (9%) of samples with a median copy number of 1.2 (range 0.75–3.1). Point mutation F184Y in *Pfmdr1* was found in 39/47 (83%) of samples, whereas the remainder showed the wild type gene.

In *Pfcrt* the observed haplotype patterns covering amino acid 72–76 included C72S, V73, M74I, N75E/D, K76T (CVIET) in 99/101 (98%) of samples and CVIDT in (2/101) 2%. None of the parasites strains contained the wild type *pfcrt* gene.

Six point mutations in *pfdhfr* and five mutations in *pfdhps* were assessed. Quadruple mutations at N51I, C59R, S108N and I164L of the *pfdhfr* gene were found in 24/101 (24%) of samples, whereas a triple mutation at N51I, C59R, and S108N was observed in 77/101 (76%) of samples. For *pfdhps*, the triple mutation at position S436A, A437G, K540E was predominant (79/86: 92%), whereas the remaining types were a triple mutation of A437G, K540N, A581G (4/86: 5%) or A437G, K540E, A581G (1/86: 1%), and the quadruple mutation of S436A, A437G, K540N, A581G (1/86: 1%), and S436A, A437G, K540E, A581G type (1/86: 1%).

Combining the different resistant markers, the most frequent pattern observed in this study was C580Y (*PfKelch*), CVIET (*Pfcrt*), N51I, C59R, S108N (*Pfdhfr*), S436A, A437G, K540E (*Pfdhps*) and a single copy number of *pfmdr1*. This combination accounted for 20/47 (42%) of all samples (Fig. 1B). In 21% (10/47) of samples a higher number of resistant genes was detected: C580Y or R539T (*PfKelch*), CVIET(*Pfcrt*), N51I, C59R, S108N, I164L (*Pfdhfr*), S436A, A437G, K540E/N and/or A581G (*Pfdhp*s) and a single copy number of *pfmdr1*. A combination of propeller region C580Y or R539T *PfKelch* mutations and an increase in *Pfmdr1* copy number was observed in 11% (5/47) of patients.

### Typing of recurrent infections

A total of 10/101 (10%) patients presented to the hospital with recurrent *P. falciparum* infections. The median time after the initial presentation was 35 days (range 8 to 119 days). *Msp1*, *msp2* and *glurp* genotyping suggested that 6/10 were caused by recrudescence, thus denoting late clinical failure, whereas 4/10 had a reinfection with a different parasite strain. In patients with recurrent infection, mean (SD) multiplicity of infection was 1.28 (0.46). In the recrudescent infections, the proportion of strains with *Pfmdr1* copy number amplification was 2/6 (33%).

### Cluster analysis

**Figure 2:**
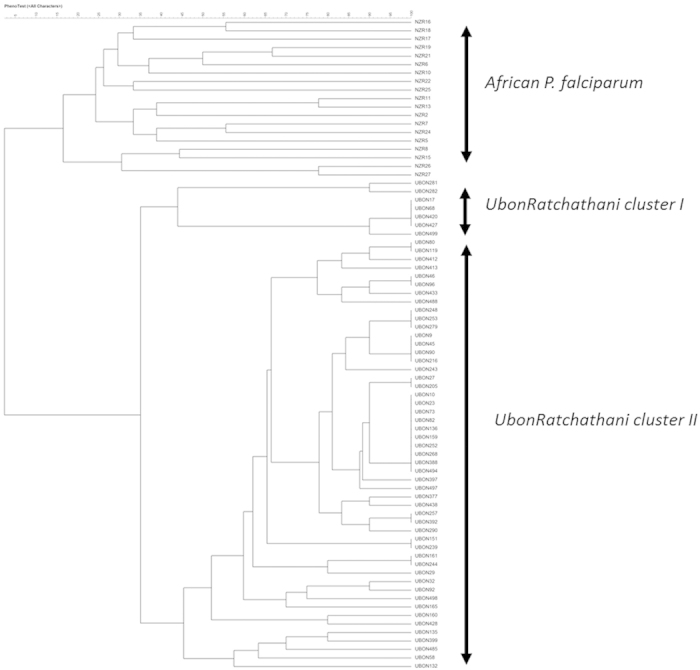
Dendrogram showing inter-strain relatedness of *P. falciparum* strains carrying the C580Y *Pfkelch* mutation obtained from patients with a first presentation of uncomplicated falciparum malaria in Buntharik district hospital in Ubon Ratchatani province in eastern Thailand. Complete microsatellite typing was successful in 57/65 (88%) of parasite strains with the C580Y mutation. Microsatellite types were compared to 20 typed strains from Guinea^16^. Cluster analysis was based on typing of 9 microsatellites using genetic similarity indexes obtained by unweighted pair group method arithmetic averages (UPGMA). The analysis revealed 2 separate clusters within the Ubon Ratchathani isolates.

Microsatellite typing and subsequent cluster analysis by UPGMA was performed on all 65 parasite strains containing the C580Y *Pfkelch* mutation obtained from patients at the moment of first presentation. Complete typing was successful in 57 samples, whereas in the remainder the quantity of parasite DNA was too limited. Results are presented as a dendrogram in Fig. 2. Previous published microsatellite genetic patterns of 20 *P. falciparum* strains from Guinea^16^ were included in the dendrogram as an outgroup. The dendrogram indicated that the *P. falciparum* strains obtained from patients in Ubon Ratchathani were closely related and could be divided into 2 clusters. The *P. falciparum* from Guinea clearly diverged from the Thai isolates.

## Discussion

Our results suggest that the vast majority of *P. falciparum* strains causing the recent outbreak of falciparum malaria in Ubon Rachathani in eastern Thailand is artemisinin resistant, as defined by the presence of mutations in the *PfKelch* gene on chromosome 13 (K13), which have a proven association with artemisinin resistance. Single point mutations in the so called propeller region of this gene, coding for a Kelch protein domain with a β-propeller tertiary structure, are strongly associated with the slow-clearance artemisinin resistant phenotype^12^. Confirmation of K13 as the major locus conferring *P. falciparum* resistance has been provided by Genome-Wide Association Studies (GWAS) and transfection studies inducing genetic modification of the K13 locus of the parasite^17^,^18^,^19^. The vast majority of K13 mutations in patients from Ubon Ratchatani were in position 580 or 539, which is similar to the K13 mutations observed in Western Cambodia and Southern Laos^12^. In addition, the majority of strains in our sample carried resistant markers for chloroquine and sulphadoxin-pyrimethamine. *Pfmdr1* gene amplification was rare, suggesting sustained susceptibility to mefloquine^13^. Most parasite strains showed the *Pfmdr1* F186Y mutation, which was also highly prevalent (78%) in *P. falciparum* strains obtained from Western Cambodia in 2008, when dihydroartemisinin-piperaquine was first-line therapy^20^. Selection of this allele has been described in the context of amodiaquine drug pressure, but not for mefloquine or lumefantrine^21^,^22^. Two major patters of multiple molecular drug resistance markers were observed, accounting for over half of the parasite strains.

Similar patterns of drug resistance markers have been found in southern Laos and western Cambodia. This suggests that the outbreak in the southern provinces in Laos and Ubon Ratchatani have the same origin. However, independent emergence of specific K13 mutations has been described^23^. Genetic epidemiology assessing the relatedness of parasite strains between the different parasite populations through genetic barcoding or microsatellite typing would provide important additional information on the origin and spread of the current increase in *P. falciparum* transmission in the adjacent provinces in Thailand and Laos.

It is also very important to know whether artemisinin resistance has contributed to the current outbreak. Current levels of artemisinin resistance do not translate into increased treatment failure with ACT as long as there is sustained sensitivity to the partner drug. The majority of strains in our study showed a single *Pfmdr1* copy number which should translate into sustained efficacy of artesunate-mefloquine, which is the first line treatment in Ubon Ratchatani. With passive follow-up recrudescent infection was observed in only 6/101 (6%) of cases of which 2 patients carried parasites with multiple copy numbers of *Pfmdr1*. Since there was no active follow-up the actual number of recrudescent infections might have been higher, and a more formal efficacy study is warranted.

On the Laos side of the border artemether-lumefantrine is first line treatment. There are no strong molecular correlates for lumefantrine resistance, but selection for *Pfmdr1* N86 and D1246 (both wild type) has been observed in patients exposed to artemether-lumefantrine, whereas 86Y and 1246Y are more prevalent after amodiaquine pressure^22^. Although parasites in the current study carried wild type *Pfmdr1* (N86 and D1246), there have been no reports of increased treatment failure rates with artemether-lumefantrine in southern Laos. The therapeutic efficacy studies in Southern Laos show that cure rates with artemether-lumefantrine rates have remained high since 2005. Continued monitoring will be crucial, since in the presence of artemisinin resistance, ACTs are prone to fall also to partner drug resistance^24^.

Also in the absence of ACT treatment failure, artemisinin resistance can increase malaria transmission through an increase in the proportion and duration of gametocyte carriage^12^. An increase in these sexual forms of the parasite can contribute to an increase in transmission. The close relatedness of the parasite strains in the cluster analysis and the very high prevalence of the K13 artemisinin resistance marker (93%) suggest recent expansion of artemisinin resistant strains. A limited number of founder populations of artemisinin resistant parasite strains has been described previously in Cambodia, where artemisinin resistance was first reported^25^. Relatedness between strains is less than observed in previous microsatellite genotyping studies in artemisinin resistant *P. falciparum* in Pailin in western Cambodia. This earlier study showed very limited genetic diversity, with single clones identified in multiple patients^26^. Yet, malaria transmission in Pailin, as in most areas where artemisinin resistance has been described, is very low and the current outbreak of artemisinin resistant falciparum malaria might reflect a next phase in the increasing problem of antimalarial drug resistance with rapid expansion of a multitude of resistant strains with apparent good biological fitness.

However, increased exposure to the disease is an alternative explanation for the recent outbreak. There is anecdotal information of increased cross-border activities involving wood-cutting and other forest activities. An increase in vectorial capacity is another possibility, but has not been reported. Access to treatment is unlikely to have declined over the last years, but inadequate treatment with incomplete treatment courses, substandard medication, or artemisinin monotherapies are potential additional contributors. Counterfeit and monotherapy artemisinins have been identified as an important problem in the region in the past^27^,^28^. However, since then artemisinin monotherapies have been withdrawn from the market in all countries of the GMS, and the problem of counterfeit antimalarials has reduced^29^. It will be crucial to investigate the causes of the current outbreak in more detail, since the appropriate response will depend on the contributing causes.

Our study has several shortcomings. The sample size is relatively small compared to the extent of the current outbreak. However, given the wide catchment area of the hospital where the study was performed, we think that our sample does represent the circulating parasite strains in the area. Therapeutic efficacy of artesunate-mefloquine was only assessed through passive follow-up of patients, so that the number of recrudescent infections could have been substantially higher. The study only had access to samples from the Thai side of the border, so that the relationship with the current outbreak in southern Laos could not be formally assessed.

In conclusion, this study describes for the first time an important outbreak of artemisinin resistant falciparum malaria in an area with previously very low transmission. Extended epidemiological studies, including genetic epidemiology, are needed to reveal the origins and spread of the outbreak. Close monitoring of ACT efficacy is crucial. This information should then guide the choice and target of additional interventions needed to counter this serious threat for malaria control in Thailand and beyond.

## Material and Methods

### Study site and patients

The study site was Buntharik district hospital (14°45′24″N 105°24′41″E) in Ubon Rachathani and recruitment was from June till October 2014. The catchment area of the hospital includes amphoes Buntharik, Na Chaluai, Pibonmungsahar and Sirinthorn, all in Ubon Ratchathani, and bordering Champasak province in southern Laos PDA. The total area covers 1402 square kilometer, with 120 villages and a population of 92,321. Ethical approval for the study was obtained from the ethical review board of the Faculty of Tropical Medicine, Mahidol University (TMEC 12–046). Patients presenting between June to October 2014 with symptomatic falciparum malaria diagnosed by rapid diagnostic test or peripheral blood microscopy were included in the study after written informed consent was obtained from all subjects. The collecting, processing, and handling of venous blood samples were carried out in accordance with the Appropriate Technology in Health (PATH) guidelines^30^. An EDTA anticoagulated blood sample was drawn before start of therapy and kept at −20 °C until DNA extraction at the Faculty of Tropical Medicine, Mahidol University, Bangkok. DNA purification was performed using a QIAgen kit (QIAgen, Germany) according to the manufacturer’s instructions.

### Assessment of *P. falciparum* resistance genes

Point mutations in the *PfKelch* gene were assessed by nested PCR amplification covering the full length of the gene (2181bp including one exon) followed by sequencing of the gene by ABI Sequencer (Macrogen Inc, South Korea). The sequencing results were then aligned against the *PfKelch* gene of reference strain 3D7 (putative 9PF13_0238 NCBI Reference Sequence (3D7): XM_001350122.1). Analysis was performed with Bioedit software (Abbott, CA, USA).

Polymorphisms in the genes *P. falciparum* chloroquine resistance transporter (*Pfcrt*) and *P. falciparum* multi drug resistance transporter (*Pfmdr1*) were assessed by nested PCR and direct gene sequencing using previously described primers and according to previously published methods^31^.

The *P. falciparum dihydrofolate reductase* (*pfdhfr*) and *dihydropteroate synthase (pfdhps)* genes were amplified from the DNA template using nested PCR. A PCR-restriction fragment length polymorphism assay was then used to assess the mutations at position *pfdhfr* 57, 58, 61, 117, 156, and 173 and *pfdhps* 382, 383, 512, 553, and 585, as described previously^32^. Digestion fragments were analyzed on a 3% agarose gel. For quality control a random third of all PCR products were sent for DNA sequencing at a commercial laboratory (Macrogen Inc, South Korea).

*Pfmdr1* copy number was quantified using Taqman real time PCR on a Corbett Rotor-Gene™ Q (Corbett Research, Australia). Primers and probes have been described previously^13^. Amplification was performed in triplicate on a total volume of 10 μL as multiplex PCR using Quantitec Multiplex PCR no ROX (QIAgen, Germany). Every amplification run contained 9 replicates of calibrators and triplicates without template as negative controls. *β-tubulin* served as an internal standard for the amount of sample DNA added to the reactions. Copy numbers were calculated using the formula: copy number= **2**^**−ΔΔCt**^; with **ΔΔ C**_**t**_denoting the difference between Δ C_t_ of the unknown sample and Δ C_t_ of the reference sample.

### Categorising recurrent infections

Although there was no active follow up after treatment, patients were advised to return to the hospital if they experienced fever. With this passive follow-up a total of 10 patients were found to have recurrent parasitaemia by microscopy. From these patients a blood sample was collected for *P. falciparum* genotyping and compared with the genotype of the primary infection. For this, nested PCR amplification was performed as described previously based on the genes for surface proteins of the *Plasmodium falciparum* merozoite (MSP-1 and MSP-2) and the glutamate-rich protein (GLURP)^33^. Analyses of the polymorphic patterns were compared between samples obtained before and after treatment to distinguish reinfection from recrudescence.

### Genetic variation and cluster analysis

We hypothesized that if the upsurge in falciparum malaria cases in Ubon Ratchathani is caused by an outbreak of artemisinin resistant parasite strains, these strains would be more closely related than expected by chance. In order to assess relatedness between infecting parasite strains microsatellite genotyping was performed. Nine *P. falciparum* microsatellite markers of tri-nucleotide repeats, named PFPK2, ARA2, TA42, TA1, TA81, Polya, TA60, Pfg377, TA87, were assessed using semi-nested PCR as described previously^34^. The length of PCR generated products were measured in comparison to internal size standards (Genescan 500 LIZ) on an ABI 3100 Genetic analyzer (PE Applied Biosystems), using Genescan and Genotyper software (Applied Biosystems) to measure allele length and quantify peak heights. Samples that amplified poorly for particular loci (maximum peak height <300 fluorescent units) were rerun. Negative control samples containing no template were included in each amplification run to rule out cross-contamination. A subset of 10 samples was analysed in triplicate to confirm intra-assay consistency.

Genetic similarity between isolates was analyzed and a dendrogram was constructed based on the nine microsatellite markers using an unweighted pair group method with arithmetic average (UPGMA) using BioNumerics software version 7.5 (Applied Maths N.V., Belgium).

## Additional Information

**How to cite this article**: Imwong, M. *et al.* An outbreak of artemisinin resistant falciparum malaria in Eastern Thailand. *Sci. Rep.* **5**, 17412; doi: 10.1038/srep17412 (2015).
